# A Simplified Fractional Order PID Controller’s Optimal Tuning: A Case Study on a PMSM Speed Servo

**DOI:** 10.3390/e23020130

**Published:** 2021-01-20

**Authors:** Weijia Zheng, Ying Luo, YangQuan Chen, Xiaohong Wang

**Affiliations:** 1 School of Mechatronic Engineering and Automation, Foshan University, 33 Guangyun Road, Foshan 528225, China; z.wj08@mail.scut.edu.cn; 2 Department of Mechanical Science and Engineering, Huazhong University of Science and Technology, 1037 Luoyu Road, Wuhan 430074, China; 3 School of Engineering, University of California, Merced, 5200 North Lake Road, Merced, CA 95340, USA; ychen53@ucmerced.edu; 4 School of Automation Science and Engineering, South China University of Technology, 381 Wushan Road, Guangzhou 510641, China; xhwang@scut.edu.cn

**Keywords:** fractional order PID control, PMSM, frequency-domain control design, optimal tuning

## Abstract

A simplified fractional order PID (FOPID) controller is proposed by the suitable definition of the parameter relation with the optimized changeable coefficient. The number of the pending controller parameters is reduced, but all the proportional, integral, and derivative components are kept. The estimation model of the optimal relation coefficient between the controller parameters is established, according to which the optimal FOPID controller parameters can be calculated analytically. A case study is provided, focusing on the practical application of the simplified FOPID controller to a permanent magnet synchronous motor (PMSM) speed servo. The dynamic performance of the simplified FOPID control system is tested by motor speed control simulation and experiments. Comparisons are performed between the control systems using the proposed method and those using some other existing methods. According to the simulation and experimental results, the simplified FOPID control system achieves the optimal dynamic performance. Therefore, the validity of the proposed controller structure and tuning method is demonstrated.

## 1. Introduction

Recently, fractional calculus has attracted increasing interest in various fields of science and engineering [1,2,3,4]. Fractional calculus is a generalization of the traditional integral and differential operators from integer order to real number order [5,6,7,8]. Thus, it has a larger feasible scope and greater flexibility in the system modeling and controller design methodology than the classical integer order one [9,10,11]. Fractional control has aroused theoretical and practical interest in the control community. Different kinds of fractional order controllers and tuning methods have been introduced and studied [12,13,14].

The fractional order proportional-integral-derivative (FOPID) controller has the tunable integral and differential orders, creating the possibility to provide better control performance [15]. However, the design of the FOPID controller is also more difficult. Generally, the tuning methods of the FOPID controller can mainly be divided into the analytic design methods and the optimization methods. The classic frequency-domain method is a typical analytic design method for the FOPI/D controller. Applying this method, three equations can be derived from three frequency-domain specifications [16], according to which the controller parameters can be calculated. However, with only three specifications, this method may not be directly used to design the FOPID controller with five degrees of freedom. On the other hand, the optimization design methods are based on iterative optimization [17,18]. Applying the optimization methods, the FOPID controller parameters are obtained by optimizing an objective function characterizing the performance of the control system, under the constraints corresponding to specific design requirements, such as the system stability and sensitivity [19]. Thus, an optimal FOPID controller can be obtained using the optimization method, but the optimization process requires sufficient time and computing capability.

In our previous work, an analytic design method was proposed for the FOPID controller, according to the linear relation between the controller parameters [20]. On this basis, an improved FOPID controller is proposed in this paper, building the nonlinear relation between the integral gain $K_{i}$ and derivative gain $K_{d}$, with a changeable coefficient. The optimal coefficient is modeled using the numerical fitting method, based on its optimal distribution with regard to the plant model characteristics and design specifications. With the estimated model, the parameters of the optimal FOPID controller can be calculated analytically according to the design specifications. Compared with our previous work, the improved FOPID controller proposed in this paper can be applied to a larger scope of plant models and design specifications because a more sophisticated relation between the controller parameters is adopted.

A case study of the proposed controller on the PMSM speed control is provided. The robustness to the gain variations, step response performance, and anti-load disturbance performance of the FOPID control system are tested by simulations and experiments. Comparisons are performed between the control systems using the proposed controller and those using some existing FOPID controllers. The advantages of the proposed method are demonstrated by simulation and experimental results.

The contributions of this paper mainly include: (1) The relations among the FOPID controller parameters are reasonably defined with a changeable coefficient, obtaining a simplified FOPID controller structure, but a complete P&I&D tunability. (2) The estimation model of the optimal relation coefficient between the controller parameters is built, realizing the optimal estimation of the fractional orders and the subsequent analytical calculation of the remaining parameters of the controller.

The paper is organized as follows: The simplified FOPID controller and the corresponding tuning method are proposed in Section 2. The estimation model of the optimal relation coefficient is discussed and established in Section 3. In Section 4 and Section 5, the application of the improved FOPID controller to the PMSM speed control is studied. The robustness and dynamic performance of the control system using the simplified FOPID controller are verified by simulations and experiments. The conclusion is presented in Section 6.

## 2. Simplified FOPID Controller

The FOPID controller can be represented as (1),

(1) $$C\left(s\right)=K_{p}\left(1+\frac{K_{i}}{s^{\lambda }}+K_{d}s^{\mu }\right),$$

where $K_{p}$, $K_{i}$, and $K_{d}$ represent the gains of the proportional, integral, and derivative components, respectively; λ and μ are the real number orders with 0<λ<2 and 0<μ<2.

**Figure 1:**
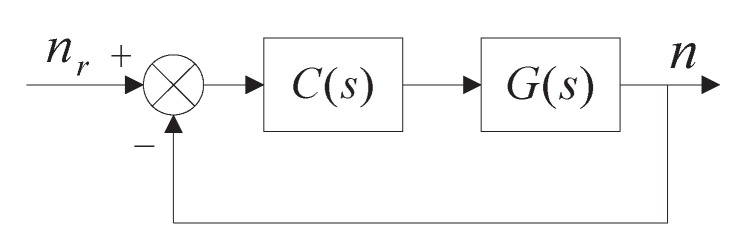
The closed-loop control system.

The typical unit negative feedback control system can be represented as Figure 1, where G(s) and C(s) are the plant and controller, respectively, and $n_{r}$ and *n* are the reference and output signals, respectively. The classic frequency-domain method depends on three specifications, i.e., the gain crossover frequency $\omega_{c}$, the phase margin $\varphi_{m}$, and the slope of the phase at $\omega_{c}$ [21], yielding,

(2) $$|C\left(j\omega_{c}\right)G\left(j\omega_{c}\right)|=1,$$

(3) $$\mathrm{Arg}\left[C\left(j\omega_{c}\right)\right]+\mathrm{Arg}\left[G\left(j\omega_{c}\right)\right]=-\pi +\varphi_{m},$$

(4) $$\frac{d[\mathrm{Arg}[C(j\omega )G(j\omega )]]}{d\omega }{|}_{\omega =\omega_{c}}=0,$$

Therefore, the parameters of the FOPI or FOPD controllers can be calculated according to these specifications. However, five pending parameters of the FOPID controller cannot be solved according to only three equations.

To solve this problem, a relation between $K_{i}$ and $K_{d}$ is proposed as (5),

(5) $$K_{d}=\frac{1}{aK_{i}},$$

where *a* is a changeable coefficient. The dynamic characteristics of the FOPID controller, e.g., the overshoot and oscillation of the step response, are affected by the fractional orders λ and μ. Taking advantage of a simple assumption [22], a relation between λ and μ is proposed as (6),

(6) λ=μ.

Thus, the FOPID controller is converted into a simplified form,

(7) $$C\left(s\right)=K_{p}\left(1+\frac{K_{i}}{s^{\lambda }}+\frac{1}{aK_{i}}s^{\lambda }\right).$$

The amplitude and phase of the simplified FOPID controller can be obtained,

(8) $$\left|C(j\omega )\right|=K_{p}\sqrt{P{\left(\omega \right)}^{2}+Q{\left(\omega \right)}^{2}},$$

(9) $$\mathrm{Arg}\left[C\left(j\omega \right)\right]=\arctan\left(\frac{Q(\omega )}{P(\omega )}\right),$$

where:

(10) $$P\left(\omega \right)=1+K_{i}\omega^{-\lambda }\cos\left(\frac{\pi }{2}\lambda \right)+\frac{1}{aK_{i}}\omega^{\lambda }\cos\left(\frac{\pi }{2}\lambda \right),$$

(11) $$Q\left(\omega \right)=\frac{1}{aK_{i}}\omega^{\lambda }\sin\left(\frac{\pi }{2}\lambda \right)-K_{i}\omega^{-\lambda }\sin\left(\frac{\pi }{2}\lambda \right).$$

If $\omega_{c}$ and $\varphi_{m}$ are given as the design specifications, substituting (9) into (3) yields,

(12) $$\arctan\left(\frac{Q(\omega_{c})}{P(\omega_{c})}\right)+\mathrm{Arg}\left[G\left(j\omega_{c}\right)\right]=-\pi +\varphi_{m}.$$

Assuming that the coefficient *a* has been determined, denoting *T* as $\tan(-\pi +\varphi_{m}-\mathrm{Arg}\left[G\left(j\omega_{c}\right)\right])$, an equation about $K_{i}$ and λ can be obtained,

(13) $$s_{1}{K_{i}}^{2}+s_{0}K_{i}-\frac{1}{a}=0,$$

where:

(14) $$s_{1}=\frac{T{\omega_{c}}^{-\lambda }\cos\left(\frac{\pi }{2}\lambda \right)+{\omega_{c}}^{-\lambda }\sin\left(\frac{\pi }{2}\lambda \right)}{{\omega_{c}}^{\lambda }\sin\left(\frac{\pi }{2}\lambda \right)-T{\omega_{c}}^{\lambda }\cos\left(\frac{\pi }{2}\lambda \right)},$$

(15) $$s_{0}=\frac{T}{{\omega_{c}}^{\lambda }\sin\left(\frac{\pi }{2}\lambda \right)-T{\omega_{c}}^{\lambda }\cos\left(\frac{\pi }{2}\lambda \right)}.$$

Substituting (9) into (4), another equation about $K_{i}$ and λ is obtained,

(16) $$\begin{array}{cc} & \frac{\lambda {\omega_{c}}^{\lambda -1}}{aK_{i}}\sin\left(\frac{\pi }{2}\lambda \right)+\frac{2\lambda }{a\omega_{c}}\sin\left(\lambda \pi \right)+\frac{M}{{\omega_{c}}^{2\lambda }}{K_{i}}^{2}+\frac{M{\omega_{c}}^{2\lambda }}{a{K_{i}}^{2}}+\frac{2M}{a}\cos\left(\lambda \pi \right)\\ & +\frac{2M{\omega_{c}}^{\lambda }}{aK_{i}}\cos\left(\frac{\pi }{2}\lambda \right)+\lambda {\omega_{c}}^{\lambda -1}K_{i}\sin\left(\frac{\pi }{2}\lambda \right)+\frac{2M}{{\omega_{c}}^{\lambda }}\cos\left(\frac{\pi }{2}\lambda \right)+M=0. \end{array}$$

where:

(17) $$M=\frac{d[\mathrm{Arg}[G(j\omega )]]}{d\omega }{|}_{\omega =\omega_{c}}.$$

The integral gain $K_{i}$ and order λ can be calculated by solving (13) and (16), and then, the proportional gain $K_{p}$ can also be calculated by solving (2). Thus, if *a* is determined, all the parameters of the simplified FOPID controller can be calculated according to the design specifications.

## 3. Estimation Model Establishment

According to the proposed tuning method, the coefficient *a* should be determined before the calculation of the FOPID controller parameters. Thus, in order to improve the control performance, the distribution of the optimal *a* should be studied. In this paper, we concentrate on the third-order plant model described by (18),

(18) $$G\left(s\right)=\frac{K}{s^{3}+\tau_{1}s^{2}+\tau_{2}s},$$

where *K*, $\tau_{1}$, and $\tau_{2}$ are the parameters of the plant. The estimation model of *a* is established in the hyperspace defined by the ranges of the plant model parameters ($\tau_{1}$, $\tau_{2}$) and the design specifications ($\omega_{c}$, $\varphi_{m}$). The ranges of $\tau_{1}$ and $\tau_{2}$ are determined according to the parameters of the plant models in actual applications, while those of $\omega_{c}$ and $\varphi_{m}$ are determined according to the design requirements. In this paper, the range of $\tau_{1}$ is set from 90 to 180 and that of $\tau_{2}$ is set from 6000 to 11,000. The range of the gain crossover frequency $\omega_{c}$ is set from 35 rad/s to 70 rad/s, and that of the phase margin $\varphi_{m}$ is set from $30^{\circ }$ to $60^{\circ }$, covering the design requirements of a class of motion control systems [23].

### 3.1. Optimal Samples’ Collection

Several values of $\tau_{1}$ and $\tau_{2}$ are uniformly selected from their ranges, respectively, obtaining ($\tau_{1,1}$, $\tau_{1,2}$, ..., $\tau_{1,m}$) and ($\tau_{2,1}$, $\tau_{2,2}$, ..., $\tau_{2,n}$). Since the plant model gain *K* has no influence on the estimation of *a*, it is given a fixed value. Thus, several test models can be established by combining the values of $\tau_{1}$ and $\tau_{2}$,

(19) $$\begin{array}{cc} \; & G_{i,j}\left(s\right)=\frac{K}{s^{3}+\tau_{1,i}s^{2}+\tau_{2,j}s}, \end{array}$$

where i=1,2,…,m,j=1,2,…,n. Similarly, several values of $\omega_{c}$ ($\omega_{c,1},\omega_{c,2},\ldots ,\omega_{c,p}$) and $\varphi_{m}$ ($\varphi_{m,1},\varphi_{m,2},\ldots ,\varphi_{m,q}$) are selected from their ranges to be the given design specifications.

The integral of time and absolute error (ITAE) is adopted as the loss function to evaluate the dynamic performance of the control system,

(20) $$J=\int_{0}^{\infty }t\left|e(t)\right|dt,$$

where e(t) represents the error between the reference and output signals.

**Figure 2:**
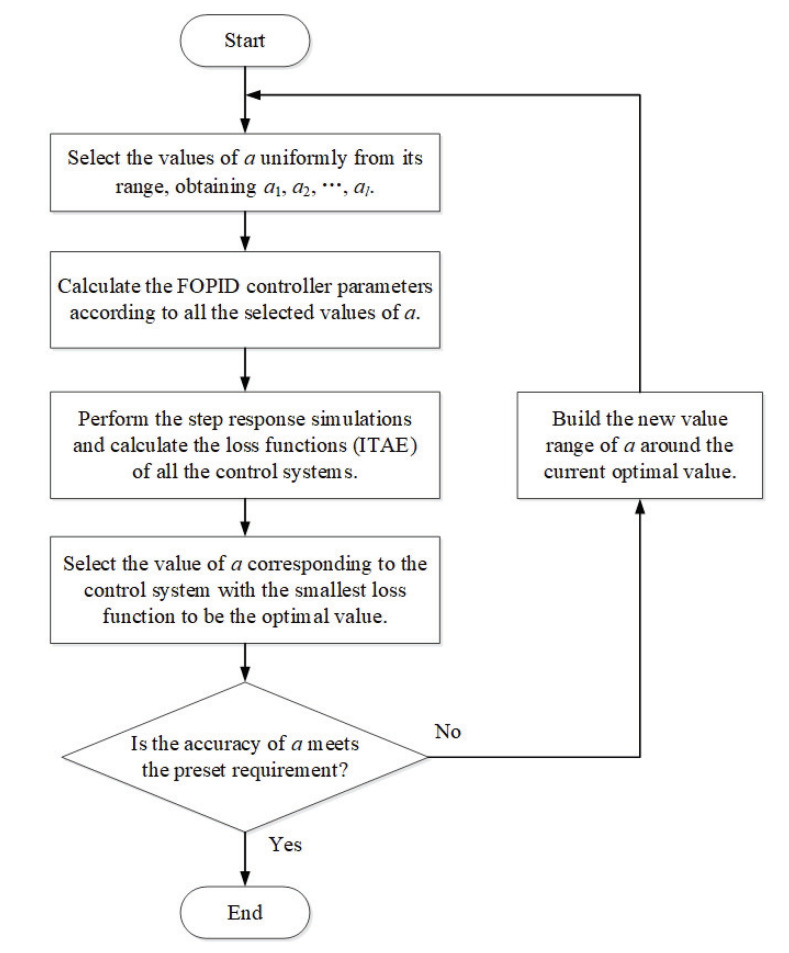
The determining process of the optimal *a*.

**Figure 3:**
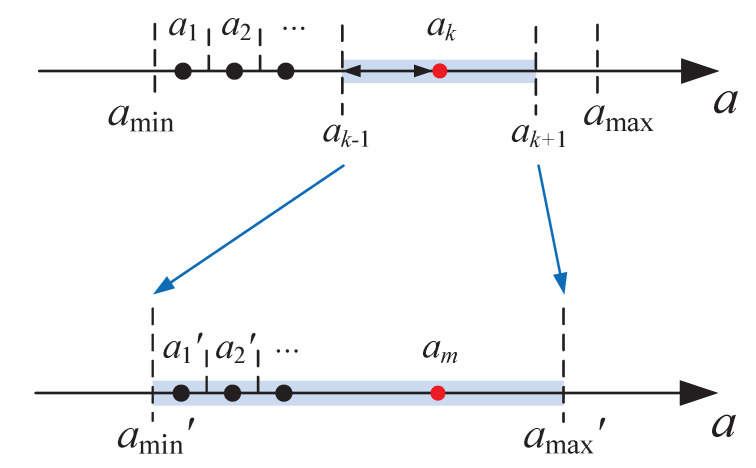
The construction of the new range of *a*.

The optimal sample of *a* for each test model ($\tau_{1}$, $\tau_{2}$) and design index ($\omega_{c}$, $\varphi_{m}$) is collected following the steps shown in Figure 2. An accuracy threshold σ is set for the search of the optimal *a*. If the value resolution of the obtained *a* is smaller than σ, this value is considered to be the optimum; otherwise, another loop of search needs to be performed in a smaller range of *a*. For example, as shown in Figure 3, if the *k*th value of *a*, $a_{k}$, is the current optimal value, but its resolution is larger than σ, namely $a_{k+1}-a_{k}>\sigma$, then a new range of *a* will be created as ($a_{k-1}$, $a_{k+1}$), in which a new optimum will be obtained.

According to the model parameter ranges, several values of $\tau_{1}$: 90, 100, ..., 180, and $\tau_{2}$: 6000, 6200, ..., 11,000, are selected to generate the test models. Similarly, several values of $\omega_{c}$: 34 rad/s, 36 rad/s, ..., 70 rad/s and $\varphi_{m}$: $30^{\circ }$, $32^{\circ }$, ..., $60^{\circ }$ are selected to be the design specifications. The initial range of *a* is from 0.001 to 500. The accuracy threshold σ is 0.001. Thus, following the steps shown in Figure 2, the optimal values of *a* corresponding to all the test models and design specifications are collected.

### 3.2. Estimation Model Establishment

Given the design specifications ($\omega_{c}$, $\varphi_{m}$), an optimal FOPID controller can be designed for a plant model G(s), according to an optimal value of *a*, which depends on the plant model characteristics $\left(\tau_{1},\tau_{2}\right)$ and design specifications $\left(\omega_{c},\varphi_{m}\right)$. The estimation model is established to approximate the distribution law of the optimal *a*.

**Figure 4:**
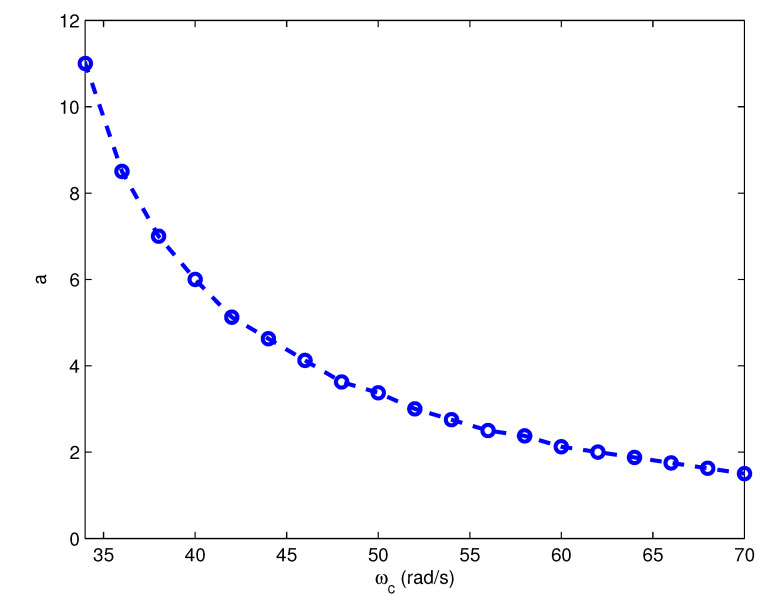
The $\omega_{c}$–*a* relation curve with $\varphi_{m}$ fixed to be $30^{\circ }$.

Firstly, the distribution of the optimal *a* for a single plant model with regard to $\omega_{c}$ and $\varphi_{m}$ is studied. Taking the test model $G_{2,5}\left(s\right)$ ($\tau_{1}=100$, $\tau_{2}=6800$) as an example, the optimal values of *a* corresponding to different given crossover frequencies $\omega_{c}$ and a fixed phase margin $\varphi_{m}$ ($\varphi_{m}=30^{\circ }$) are selected and plotted as the $\omega_{c}$–*a* relation curve in Figure 4. According to Figure 4, the distribution of the optimal *a* can be approximated as a curve.

**Figure 5:**
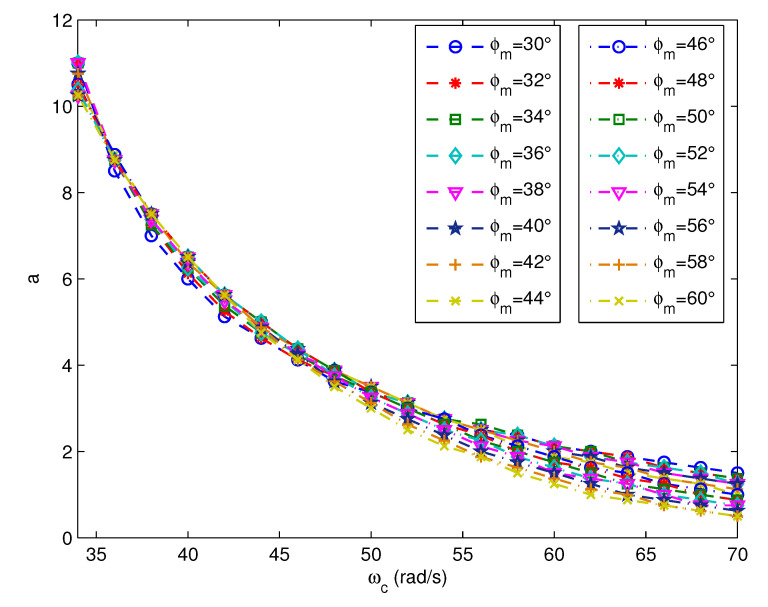
The $\omega_{c}$–*a* relation curves correspond to different $\varphi_{m}$.

The $\omega_{c}$–*a* relation curves of different $\varphi_{m}$ for test model $G_{2,5}\left(s\right)$ are plotted in Figure 5. It can be seen that the $\omega_{c}$–*a* relation curves corresponding to different $\varphi_{m}$ are close to each other. Thus, an assumption is adopted to simplify the analysis, i.e., the difference between the $\omega_{c}$–*a* relation curves corresponding to different $\varphi_{m}$ can be ignored. Therefore, for the same plant model, the optimal value of *a* is assumed to be only determined by $\omega_{c}$.

**Figure 6:**
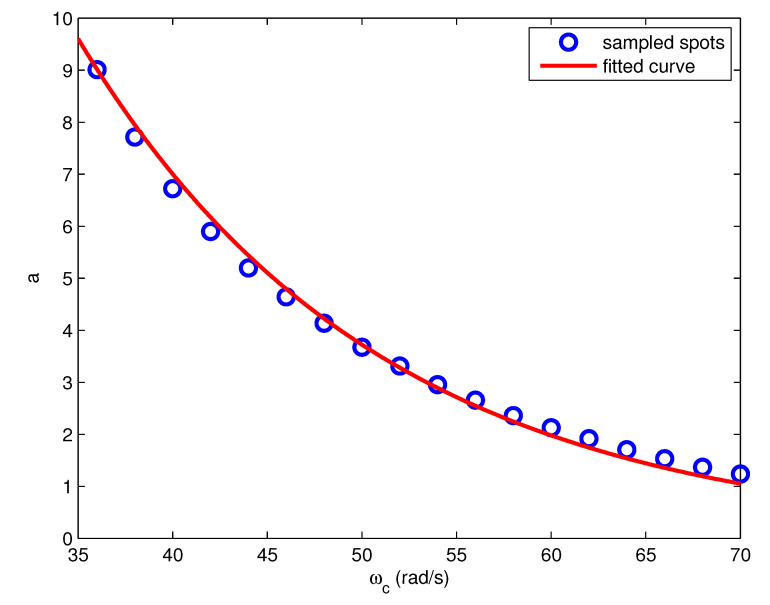
The $\omega_{c}$–mean *a* relation and fitting curve of the test model $G_{2,5}\left(s\right)$.

Adopting the simplifying assumption, an estimation model needs to be built for the mean values of the optimal *a*. The $\omega_{c}$–mean *a* relation corresponding to $G_{2,5}\left(s\right)$ is plotted as data spots in Figure 6.

It can be seen that the mean *a* values with regard to $\omega_{c}$ obey an obvious distribution law, which can be described by an exponential function,

(21) $$a=A\left(\tau_{1},\tau_{2}\right)e^{B\left(\tau_{1},\tau_{2}\right)\omega_{c}},$$

where *A* and *B* are the coefficients determined by the model parameters $\tau_{1}$ and $\tau_{2}$. The values of *A* and *B* can be obtained using the numerical fitting methods. The fitting function is plotted as the red curve in Figure 6. Fitting the $\omega_{c}$–mean *a* relations of all the plant models, the values of *A* and *B* corresponding to different plant models: $A_{i,j}$ and $B_{i,j}$, are obtained, where the subscript *i* corresponds to that of $\tau_{1,i}$ and the subscript *j* corresponds to that of $\tau_{2,j}$, i=1,2,…,m,j=1,2,…,n.

**Figure 7:**
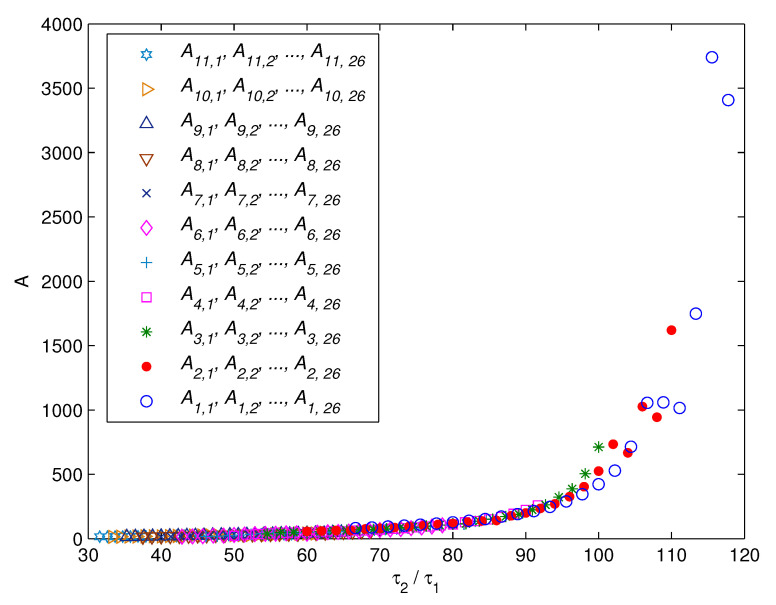
The distribution of *A* with regard to $\tau_{2}/\tau_{1}$.

Secondly, the relation between the coefficient *A* and the model parameters ($\tau_{1}$, $\tau_{2}$) is studied. Taking $\tau_{2}/\tau_{1}$ as the abscissa and the corresponding coefficient *A* as the ordinate, the distribution of *A* with regard to $\tau_{2}/\tau_{1}$ is plotted in Figure 7. As can be seen, the distribution of *A* with regard to $\tau_{2}/\tau_{1}$ can be approximated as a curve.

**Figure 8:**
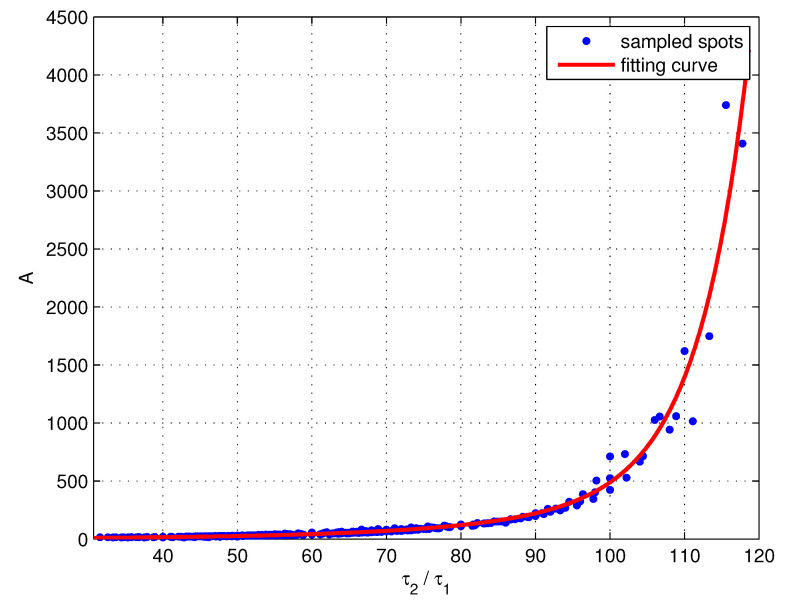
The $\tau_{2}/\tau_{1}$–*A* relation and the fitting curve.

The $\tau_{2}/\tau_{1}$–*A* relation is plotted again in Figure 8, without distinguishing the data spots corresponding to different plant models. According to the distribution of the data spots, the $\tau_{2}/\tau_{1}$–*A* relation can be fitted by a model with two exponential functions,

(22) $$A\left(\tau_{1},\tau_{2}\right)=Me^{P\frac{\tau_{2}}{\tau_{1}}}+Ne^{Q\frac{\tau_{2}}{\tau_{1}}},$$

where *M*, *N*, *P*, and *Q* are the model coefficients, which can be obtained using the numerical fitting methods. The fitting function is plotted as the red curve in Figure 8.

**Figure 9:**
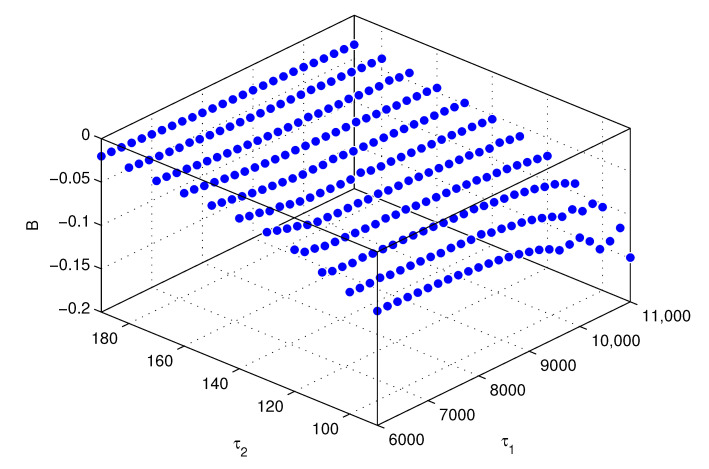
The distribution of *B* with regard to $\tau_{1}$ and $\tau_{2}$.

Thirdly, the three-dimensional distribution of coefficient *B* with regard to $\tau_{1}$ and $\tau_{2}$ is plotted in Figure 9.

Taking $\tau_{1}$ and $\tau_{2}$ as the independent variables, the ($\tau_{1}$, $\tau_{2}$)–*B* relation can be fitted by a cubic polynomial function,

(23) $$B\left(\tau_{1},\tau_{2}\right)\;=\;p_{00}\;+\;p_{10}\tau_{2}\;+\;p_{01}\tau_{1}\;+\;p_{20}{\tau_{2}}^{2}\;+\;p_{11}\tau_{2}\tau_{1}\;+\;p_{02}{\tau_{1}}^{2}\;+\;p_{30}{\tau_{2}}^{3}\;+\;p_{21}{\tau_{2}}^{2}\tau_{1}\;+\;p_{12}\tau_{2}{\tau_{1}}^{2},$$

where $p_{00}$, $p_{10}$, $p_{01}$, $p_{20}$, $p_{11}$, $p_{02}$, $p_{30}$, $p_{21}$, and $p_{12}$ are the model coefficients, which can be obtained using the numerical fitting methods. Therefore, all the coefficients of the estimation model are obtained.

## 4. Simulation Study

### 4.1. Feasible Region Study

The design flexibility of the proposed FOPID controller can be verified by studying the feasible regions of the design specifications. The feasible region of the design specifications includes the ($\omega_{c}$, $\varphi_{m}$) combinations, according to which the reasonable FOPID controller can be obtained by solving (2)–(4). To demonstrate the advantage of the proposed method, the feasible region of the simplified FOPID controller is compared with those of the FOPI and IOPID controllers.

**Figure 10:**
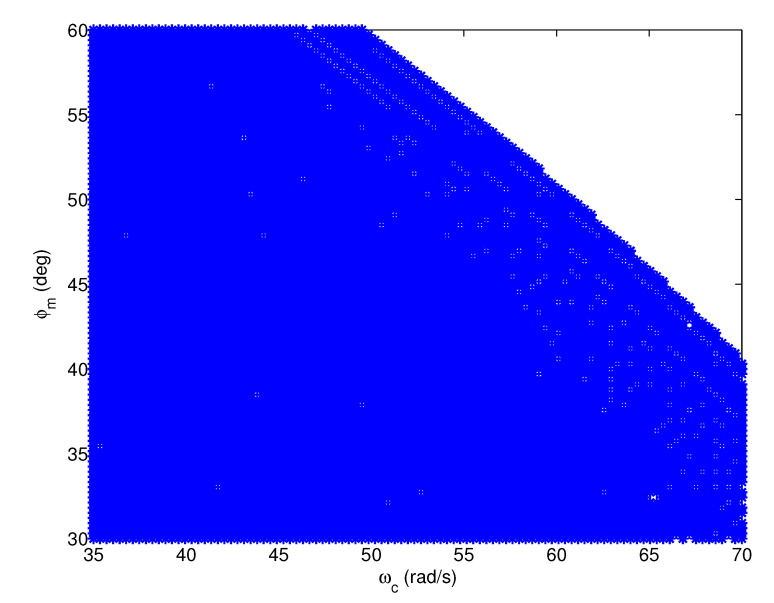
The feasible region of the FOPI controller.

**Figure 11:**
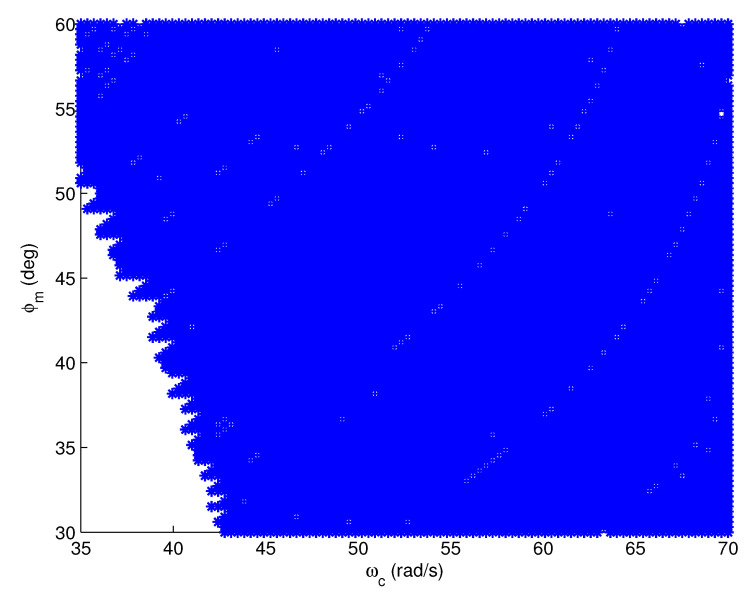
The feasible region of the IOPID controller.

**Figure 12:**
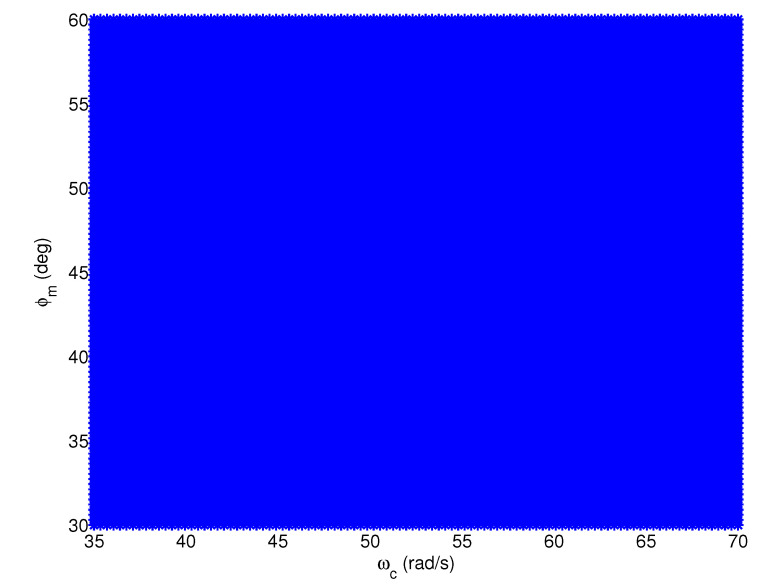
The feasible region of the FOPID controller.

Taking the test model $G_{1,26}\left(s\right)$ ($\tau_{1}$ = 90, $\tau_{2}$ = 11,000) as an example, the feasible regions of the FOPI, IOPID, and FOPID controllers are plotted in Figure 10, Figure 11 and Figure 12, respectively, where the feasible design specifications are marked in blue. According to Figure 10, if the design specifications are in the region where both $\omega_{c}$ and $\varphi_{m}$ are large, we are unable to design an FOPI controller to satisfy (2)–(4) simultaneously. Similarly, according to Figure 11, we are unable to design an IOPID controller if both $\omega_{c}$ and $\varphi_{m}$ are small. In contrast, according to Figure 12, the feasible region of the FOPID controller covers the entire region of the design specifications. Therefore, the proposed FOPID controller achieves more design options and flexibility than the FOPI and IOPID controllers.

### 4.2. PMSM Speed Servo Plant

The proposed estimation model and tuning method are applied to design the FOPID controllers for a class of PMSM speed servo systems. Applying the d−q coordinates and the field-oriented control scheme, the dynamic characteristics of a PMSM can be described by the following equations,

(24) $$u_{q}=Ri_{q}+L_{q}\frac{di_{q}}{dt}+C_{e}n,$$

(25) $$\frac{GD^{2}}{375}\frac{dn}{dt}=C_{m}i_{q}-T_{L},$$

where $u_{q}$ and $i_{q}$ are the *q*-axis voltage and current, respectively, *R* is the stator resistance, $L_{q}$ is the *q*-axis stator inductance, $C_{e}$ is the induced voltage constant, *n* is the motor speed in revolutions per minute (RPM), $C_{m}$ is the torque constant, $T_{L}$ is the load disturbance torque, and $GD^{2}$ is the flywheel inertia.

In the PMSM servo system, the *q*-axis voltage is often supplied by the pulse-width modulation (PWM) inverter, whose dynamic characteristics can be approximated by a first-order filter with time constant $T_{s}$. Adopting a PI controller as the feedback controller of the *q*-axis current,

(26) $$C_{i}\left(s\right)=K_{s}\left(1+\frac{1}{T_{s}s}\right),$$

the *q*-axis voltage can be obtained as:

(27) $$u_{q}\left(s\right)=\frac{K_{s}}{T_{s}s}\left(i_{qr}\left(s\right)-i_{q}\left(s\right)\right),$$

where $i_{qr}$ is the *q*-axis reference current. Thus, according to (24), (25), and (27), the transfer function of the PMSM speed servo plant (from $i_{qr}$ to *n*) can be represented as:

(28) $$G\left(s\right)=\frac{\frac{K_{s}}{C_{e}T_{m}T_{s}T_{l}}}{s^{3}+\frac{1}{T_{l}}s^{2}+\frac{K_{s}K_{1}}{RT_{s}T_{l}}s},$$

where $T_{l}$ is the electromagnetic time constant, $T_{l}=L/R$, and $T_{m}$ is the electromechanical time constant, $T_{m}=GD^{2}R/\left(375C_{e}C_{m}\right)$. The transfer function of the PMSM speed servo plant model used in this paper is described as:

(29) $$G\left(s\right)=\frac{47,979.257}{s^{3}+127.38s^{2}+9995.678s}.$$

### 4.3. Gain Robustness Study

Taking the PMSM speed servo as the plant model, setting the design specifications as $\omega_{c}$ = 40 rad/s and $\varphi_{m}=55^{\circ }$, the optimal coefficient *a* is estimated as 9.968. Thus, the FOPID controller is obtained,

(30) $$C_{1}\left(s\right)=8.032\left(1+\frac{13.207}{s^{0.983}}+0.0076s^{0.983}\right).$$

**Figure 13:**
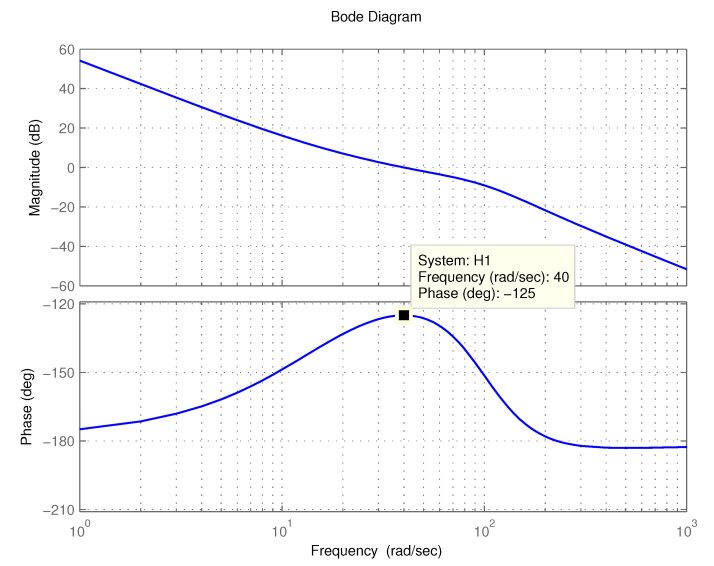
The open-loop Bode diagram of the control system.

The open-loop Bode diagram of the PMSM servo system using the FOPID controller is shown in Figure 13. It can be seen that the magnitude and phase characteristics of the control system satisfy the design specifications. The phase characteristic has zero slope at $\omega_{c}$. Thus, the systems with gain variations will have similar phase margins as the nominal system.

**Figure 14:**
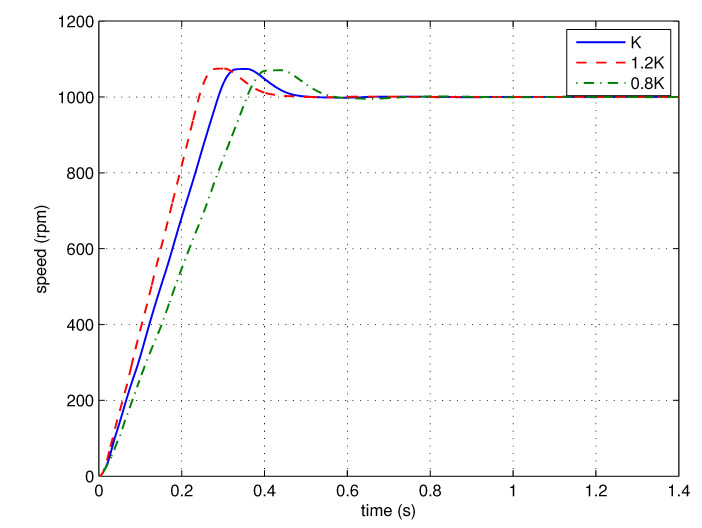
The step responses of the simplified FOPID control systems with different loop-gains (simulation).

The step response is performed to test the overshoots of the control systems with gain variations. The nominal gain of the plant is multiplied by 120% and 80% to simulate the gain variations. The step responses of the nominal system and those with gain variations are shown in Figure 14.

It can be seen that the responses of the control systems with gain variations have similar overshoots, satisfying the robustness requirement.

### 4.4. Comparisons with Some Existing Methods

An optimization-based tuning method was proposed in [24], with the sensitivity and complementary sensitivity functions introduced as the constraints. Applying this method, an optimal FOPID controller is designed for the PMSM speed control system,

(31) $$C_{3}\left(s\right)=8.896\left(1+\frac{29.815}{s^{1.299}}+0.0685s^{0.403}\right).$$

The gain crossover frequency of the obtained control system is $\omega_{c}$ = 51.6 rad/s, and the phase margin is $\varphi_{m}=50^{\circ }$. According to these design specifications, the optimal coefficient *a* is estimated as 5.047, and the FOPID controller is obtained,

(32) $$C_{4}\left(s\right)=10.451\left(1+\frac{21.017}{s^{0.991}}+0.0094s^{0.991}\right).$$

**Figure 15:**
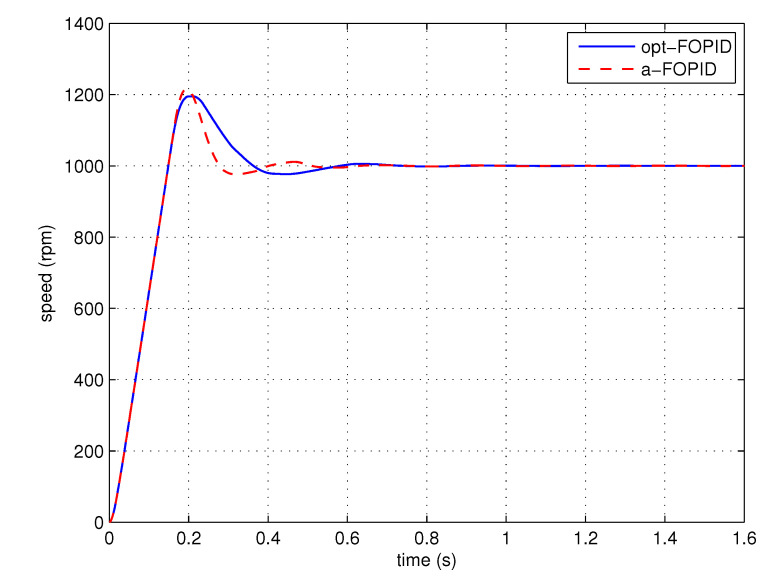
The step responses of the control systems using the opt-FOPID and a-FOPID (simulation).

The step response simulation is performed, using the optimal FOPID controller $C_{3}\left(s\right)$ (denoted as opt-FOPID) and the proposed FOPID controller $C_{4}\left(s\right)$ (denoted as a-FOPID) as the speed controllers, respectively. To guarantee a fair comparison, the two systems are made to have similar rising times. The response curves and the performance indexes are shown in Figure 15 and Table 1, respectively.

**Figure 16:**
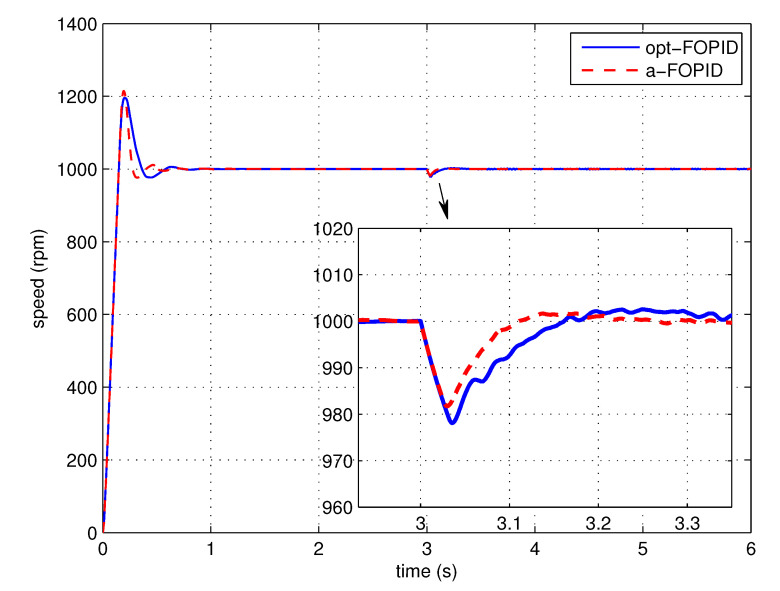
The load disturbance responses of the control systems using the opt-FOPID and a-FOPID (simulation).

The load disturbance response simulation is also performed to test the anti-load disturbance performance of the control systems. The response curves and performance indexes are shown in Figure 16 and Table 2, respectively.

According to Figure 15 and Table 1, the responses of two systems have similar overshoots, but the system using the a-FOPID has a shorter settling time. Therefore, the system using the a-FOPID achieves better step response performance. According to Figure 16 and Table 2, the response of the system using the a-FOPID has a smaller speed drop and a shorter recovery time. Therefore, the system using the a-FOPID achieves better anti-load disturbance performance.

A Bode shaping-based tuning method for the FOPID controller is proposed in [25]. Applying this method, a FOPID controller is designed for the PMSM control system,

(33) $$C_{5}\left(s\right)=7.532\left(1+\frac{49.843}{s^{1.27}}+0.0604s^{0.556}\right).$$

The gain crossover frequency of the obtained control system is $\omega_{c}$ = 41.5 rad/s, and the phase margin is $\varphi_{m}=55.7^{\circ }$. According to these design specifications, the optimal coefficient *a* is estimated as 9.128, and the FOPID controller is obtained,

(34) $$C_{6}\left(s\right)=8.362\left(1+\frac{13.628}{s^{0.986}}+0.008s^{0.986}\right).$$

**Figure 17:**
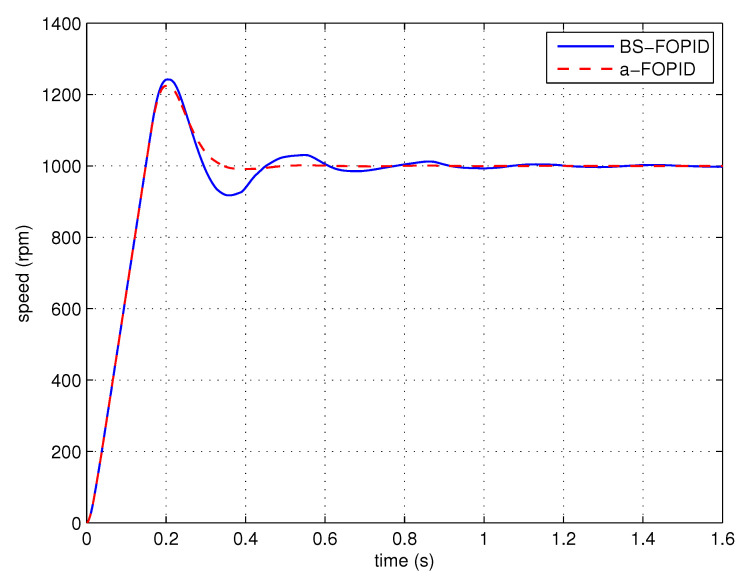
The step responses of the control systems using the Bode shaping-based (BS)-FOPID and a-FOPID (simulation).

**Figure 18:**
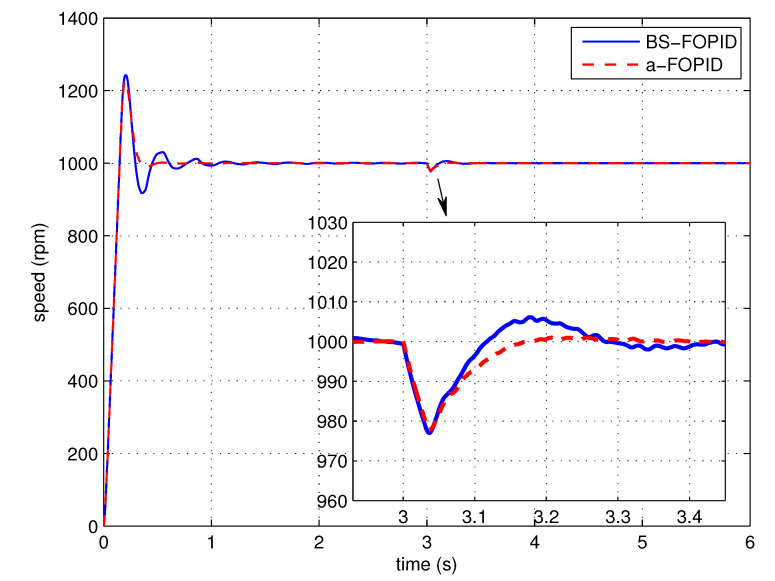
The load disturbance responses of the control systems using the BS-FOPID and a-FOPID (simulation).

Step response simulation is performed, using the Bode shaping-based FOPID controller $C_{5}\left(s\right)$ (denoted as BS-FOPID) and the proposed FOPID controller $C_{6}\left(s\right)$ (denoted as a-FOPID) as the speed controllers, respectively. The response curves and the performance indexes are shown in Figure 17 and Table 3, respectively. The load disturbance response simulation is also performed. The response curves and performance indexes are shown in Figure 18 and Table 4, respectively.

According to Figure 17 and Table 3, the response of the system using the a-FOPID has a smaller oscillation and a shorter settling time. Therefore, the system using the a-FOPID achieves better step response performance. According to Figure 18 and Table 4, the two responses have a similar speed drop and recovery time, but the response of the system using the a-FOPID has a smaller oscillation. Therefore, the system using the a-FOPID achieves better anti-load disturbance performance.

## 5. Experimental Study

**Figure 19:**
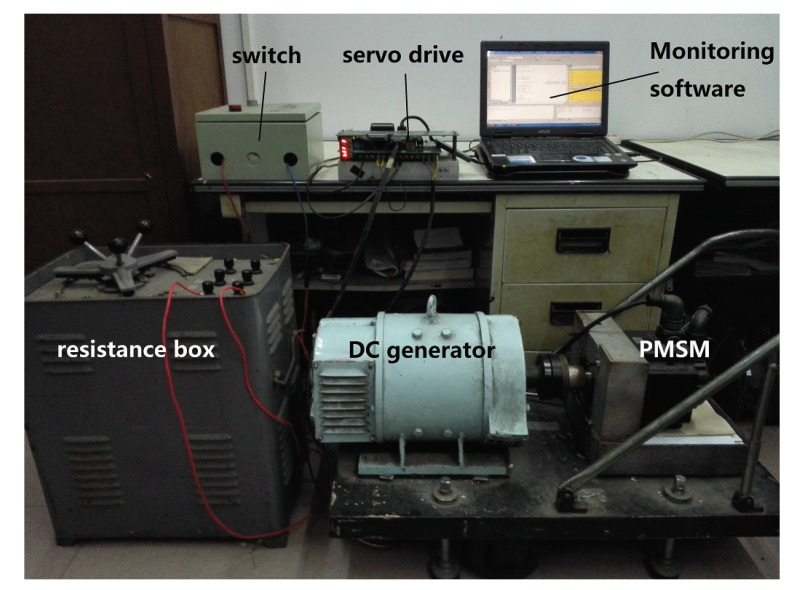
The PMSM speed control platform.

Figure 19 shows the PMSM speed control platform used in this paper. The PMSM is the model Sanyo-P10B18200BXS PMSM. In the experiments, the fractional order operator $s^{r}$ is realized by applying the impulse invariant discretization method [26].

### 5.1. Gain Robustness Study

**Figure 20:**
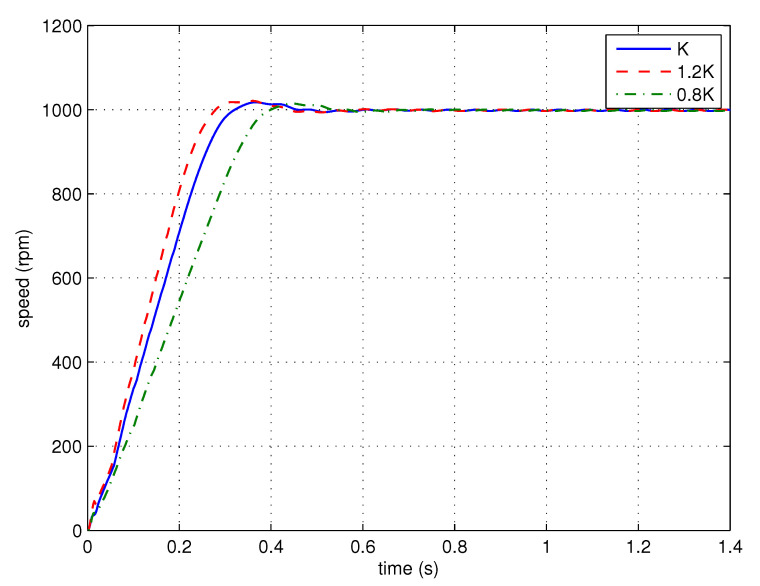
The step responses of the simplified FOPID control systems with different loop-gains (experiment).

Step response experiments are performed to test the gain robustness of the control system using the proposed FOPID controller. The proportional gain of the FOPID controller is multiplied by 120% and 80% to simulate the gain variations. The step responses of the nominal system and those with gain variations are shown in Figure 20.

According to Figure 20, similar to the simulation result, the responses of the control systems with gain variations have similar overshoots, satisfying the robustness requirement.

### 5.2. Comparisons with Some Existing Methods

**Figure 21:**
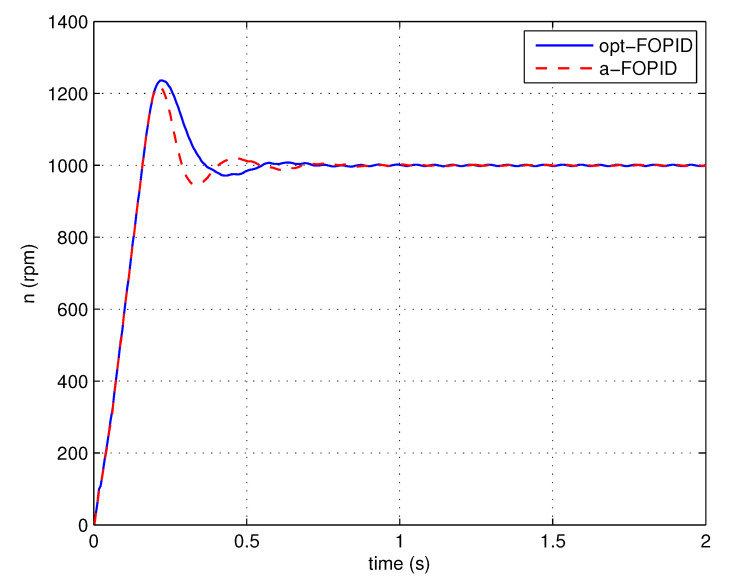
The step responses of the control systems using the opt-FOPID and a-FOPID (experiment).

**Figure 22:**
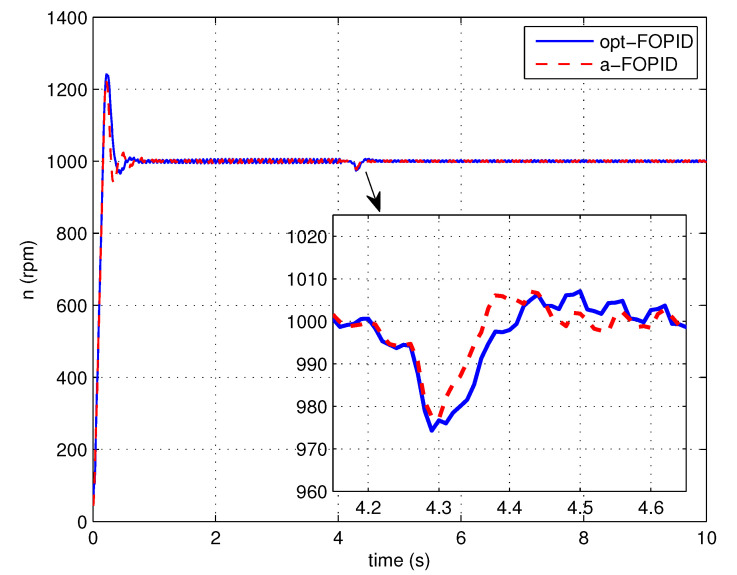
The load disturbance responses of the control systems using the opt-FOPID and a-FOPID (experiment).

Step response experiments are performed, using the optimal FOPID controller $C_{3}\left(s\right)$ (opt-FOPID) and the proposed FOPID controller $C_{4}\left(s\right)$ (a-FOPID) as the speed controllers, respectively. The response curves and the performance indexes are shown in Figure 21 and Table 5, respectively. The load disturbance response simulation is also performed to test the anti-load disturbance performance of the control systems. The response curves and performance indexes are shown in Figure 22 and Table 6, respectively.

According to Figure 21 and Table 5, similar to the simulation result, the responses of the two systems have similar overshoots, but the response of the system using the a-FOPID has a shorter settling time. Therefore, the system using the a-FOPID achieves better step response performance. According to Figure 22 and Table 6, the responses of two systems have similar speed drops, but the response of the system using the a-FOPID has a shorter recovery time. Therefore, the system using the a-FOPID achieves better anti-load disturbance performance.

**Figure 23:**
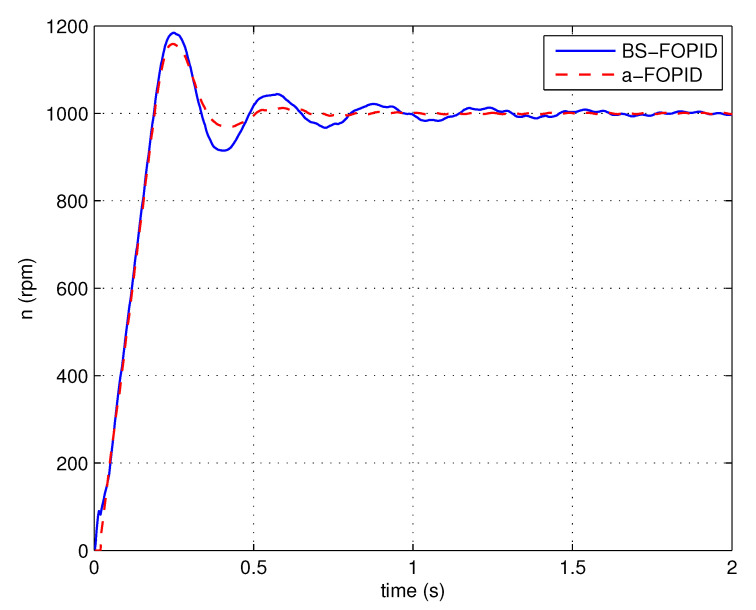
The step responses of the control systems using the BS-FOPID and a-FOPID (experiment).

**Figure 24:**
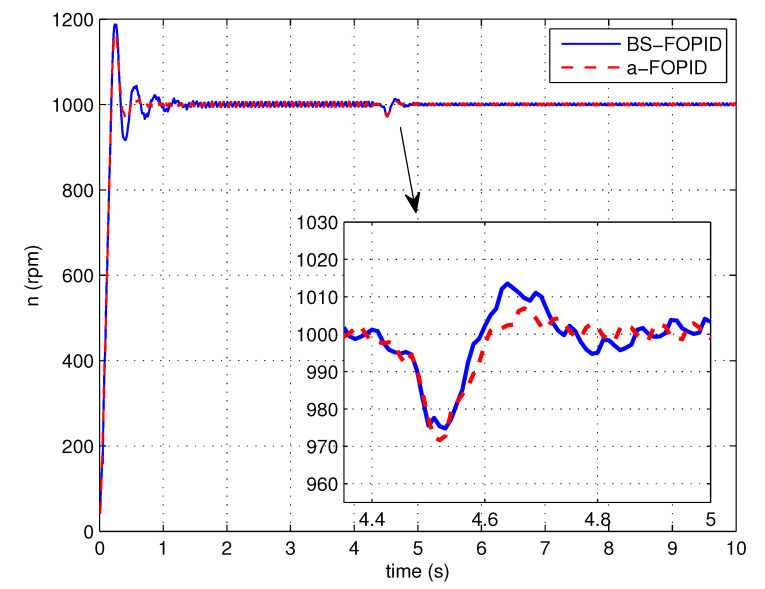
The load disturbance responses of the control systems using the BS-FOPID and a-FOPID (experiment).

Step response experiments are performed, using the Bode shaping-based FOPID controller $C_{5}\left(s\right)$ (BS-FOPID) and the simplified FOPID controller $C_{6}\left(s\right)$ (a-FOPID) as the speed controllers, respectively. The response curves and the performance indexes are shown in Figure 23 and Table 7, respectively. The load disturbance response simulation is also performed to test the anti-load disturbance performance of the control systems. The response curves and performance indexes are shown in Figure 24 and Table 8, respectively.

According to Figure 23 and Table 7, the response of the system using the a-FOPID has a smaller overshoot and a shorter settling time. Therefore, the system using the a-FOPID achieves better step response performance. According to Figure 24 and Table 8, the speed drops and recovery time of two responses are close to each other, but the response of the system using the a-FOPID has smaller oscillation. Therefore, the system using the a-FOPID achieves better anti-load disturbance performance. From the simulation and experimental results, the simplified FOPID controller achieves flexible tuning capability, sufficient robustness to gain variations, and the optimal step response performance.

## 6. Conclusions

A simplified FOPID controller is proposed by building the relations between the controller parameters. An estimation model for the optimal relation coefficient *a* is built for a class of third-order models, according to which the optimal FOPID controller controllers can be obtained analytically. An actual application of the proposed controller and tuning method on the PMSM speed servo is studied by simulation and experiments, verifying the robustness and dynamic performance of the simplified FOPID control system. The advantages of the proposed method are demonstrated by the comparisons with some other existing methods. Some issues may be studied in the future works, such as improving the relation between the fractional orders and applying the simplified FOPID controller to other classes of plants.

## Figures and Tables

**Table 1:** The step response performance indexes of the control systems using the optimal (opt)-FOPID and a-FOPID (simulation).

Control System	Settling Time (s)	Overshoot (%)
opt-FOPID	0.313	19.49
a-FOPID	0.255	21.46

**Table 2:** The anti-load disturbance performance indexes of the control systems using the opt-FOPID and a-FOPID (simulation).

Control System	Recovery Time (s)	Dynamic Speed Drop (%)
opt-FOPID	0.080	2.19
a-FOPID	0.055	1.83

**Table 3:** The step response performance indexes of the control systems using the BS-FOPID and a-FOPID (simulation).

Control System	Settling Time (s)	Overshoot (%)
BS-FOPID	0.408	24.24
a-FOPID	0.292	22.44

**Table 4:** The anti-load disturbance performance indexes of the control systems using the BS-FOPID and a-FOPID (simulation).

Control System	Recovery Time (s)	Dynamic Speed Drop (%)
BS-FOPID	0.075	2.30
a-FOPID	0.082	2.22

**Table 5:** The step response performance indexes of the control systems using the opt-FOPID and a-FOPID (experiment).

Control System	Settling Time (s)	Overshoot (%)
opt-FOPID	0.325	23.61
a-FOPID	0.273	21.91

**Table 6:** The anti-load disturbance performance indexes of the control systems using the opt-FOPID and a-FOPID (experiment).

Control System	Recovery Time (s)	Dynamic Speed Drop (%)
opt-FOPID	0.255	2.55
a-FOPID	0.195	2.30

**Table 7:** The step response performance indexes of the control systems using the BS-FOPID and a-FOPID (experiment).

Control System	Settling Time (s)	Overshoot (%)
BS-FOPID	0.452	18.45
a-FOPID	0.324	15.89

**Table 8:** The anti-load disturbance performance indexes of the control systems using the BS-FOPID and a-FOPID (experiment).

Control System	Recovery Time (s)	Dynamic Speed Drop (%)
BS-FOPID	0.265	2.52
a-FOPID	0.236	2.83

## Data Availability

The data presented in this study are available on request from the corresponding author.
